# Bibliographical

**Published:** 1868-09

**Authors:** 


### BIBLIOGRAPHICAL.

*A Manual on Extracting Teeth*, by Abraham Robertson, D. D. S., M. D.

This work "Founded on the anatomy of the parts involved in the operation; the kinds, and proper construction of the instruments to be used; the accidents liable to occur from the operation, and the proper remedies to retrieve such accidents" is the only monograph of any note upon the subject. Upon many points pertaining to the extraction of the teeth, there is, of course, a diversity of opinion; more upon the indications for the operation, than the mode of its accomplishment. The indications are not the same to different operators, but are modified somewhat by the ability of the Dentist to treat and remedy the diseases to which the teeth are subject, and that so frequently are regarded as indications for removal. Though we would differ in some respects with the author, we regard it as a very excellent work, and one that should be in every Dentist's library, and physician's too.

It is for sale by Rob't Clarke & Co., of this city. T.

*Dental Materia Medica. Compiled by* JAMES W. WHITE. *Philadelphia: Published by* SAMUEL S. WHITE.

THIS is a pretty little book of 108 pages. It fills a long felt void in Dental literature, and will prove highly useful to beginners and those who for want of opportunity, are deficient in the details of medical science, and will not prove uninteresting to the most advanced thinkers of our profession. It contains a modest preface by the compiler, a minute index, a glossary of medical terms, a table of symbols and abbreviations, weights and measures, etc., as a prelude to the carefully condensed notices of over seventy medicinal agents. The entire book gives evidence of special care in its preparation; and the information derived from it can be generally and perhaps universally relied on. W.

*Lessons in Elementary Chemistry: Inorganic and Organic. By Henry E. Roscoe, B. A., F. R. S., Professor of Chemistry in Owen's College, Manchester, New York: Wm. Wood & Co., Publishers,* 61 *Walker St.* 1868.

THIS is a beautiful little text book, well adapted to the wants of public schools and academies. Its illustrations are neatly executed, and well adapted. The publishers have done all that could be suggested in getting out the work, and the author has succeeded in condensing a great amount of useful matter into a very small space. "The metric system of weights and measures, and the centigrade thermometer scale are used throughout the work." We would like it better if the Fahrenheit scale had been used, simply because this is more generally used in America. The book is worthy of the attention of educators and students.

